# Disagreement between the results from three commercial tests for the detection of *Borrelia*-specific serum antibodies in the Netherlands associated with antibiotic treatment for Lyme borreliosis: a retrospective study

**DOI:** 10.1007/s10096-017-3037-1

**Published:** 2017-07-26

**Authors:** T. van Gorkom, K. Kremer, W. Voet, D. W. Notermans, B. J. M. Vlaminckx, S. U. C. Sankatsing, S. F. T. Thijsen

**Affiliations:** 1 Department of Medical Microbiology and Immunology, Diakonessenhuis Hospital, Bosboomstraat 1, 3582 KE Utrecht, The Netherlands; 2 Centre for Infectious Diseases Research, Diagnostics and Screening, National Institute for Public Health and the Environment (RIVM), Bilthoven, The Netherlands; 3 Department of Neurology, Diakonessenhuis Hospital, Utrecht, The Netherlands; 4 Department of Medical Microbiology and Immunology, St. Antonius Hospital, Nieuwegein, The Netherlands; 5 Department of Internal Medicine, Diakonessenhuis Hospital, Utrecht, The Netherlands

## Abstract

The diagnosis of Lyme borreliosis is challenging because of the often non-specific symptoms and persisting antibodies after infection. We investigated the diagnostic characteristics of two enzyme-linked immunosorbent assays (ELISAs) and an immunoblot for the detection of *Borrelia*-specific serum antibodies using different test strategies in individuals with and without antibiotic treatment for Lyme borreliosis. This retrospective study included healthy individuals, patients with active Lyme neuroborreliosis and patients treated for Lyme neuroborreliosis. Two ELISAs were compared: the C6 ELISA and the SERION ELISA. Equivocal and positive results were confirmed by immunoblot. We included 174 healthy individuals, of whom 27 (15.5%) were treated for Lyme borreliosis in the past, 36 patients were treated for Lyme neuroborreliosis and 27 patients had active Lyme neuroborreliosis. All the active Lyme neuroborreliosis patients were reactive in both ELISAs (100% sensitivity); less reactivity was seen in the other three groups (range 17.7% to 69.4%). The concordance between the ELISA results was high in active Lyme neuroborreliosis patients (26/27; 96.3%) and healthy individuals (131/147; 89.1%), but lower in treated healthy individuals (18/27; 66.7%) and treated Lyme neuroborreliosis patients (18/36; 50.0%) (*p* ≤ 0.005). This study showed that antibiotic treatment against Lyme borreliosis was strongly associated with discordant ELISA and test strategy results (odds ratio: 10.52; *p* < 0.001 and 9.98; *p* = 0.014, respectively) suggesting antibiotic treatment influences the pace at which the various antibodies directed to the different antigens used in both ELISAs wane. Among treated neuroborreliosis patients, the SERION ELISA stayed positive for a longer period after infection compared to the C6 ELISA. This should be taken into consideration when requesting and/or interpreting Lyme serology.

## Introduction

The recommended approach for the diagnosis of Lyme borreliosis consists of screening for *Borrelia*-specific serum antibodies with an enzyme-linked immunosorbent assay (ELISA), followed by immunoblot confirmation of equivocal or positive ELISA results [1]. The reliability of the serodiagnosis of Lyme borreliosis is influenced by various factors, including the manifestation and the duration of disease, the natural clearance of infection, antibiotic treatment, (age-specific) seroprevalence and the test characteristics, such as the antigens used [2]. Antibiotic therapy can abrogate the immune response, but the persistence of *Borrelia*-specific serum antibodies up to several years after antibiotic treatment has also been reported [3, 4]. In the Dutch population, the seroprevalence is 4–8%, but is higher in certain risk groups, such as forestry workers (20%) [5, 6]. These seropositive cases are usually asymptomatic, suggesting a cleared infection with the persistence of *Borrelia*-specific serum antibodies.

A large variety of diagnostic assays for Lyme borreliosis is available. Some assays make use of whole-cell lysates, which are mostly derived from cultured *Borrelia burgdorferi* sensu stricto, *Borrelia afzelii* or *Borrelia garinii* [7, 8]. These assays have a potential problem of higher cross-reactivity with common antigens of other micro-organisms [9]. Recombinant antigens, such as OspC, DbpA [10, 11] and especially VlsE (Vmp-like sequence) and C6 peptide are more specific [12, 13]. Although studies have compared different assays using different test strategies, these studies lacked well-defined study populations [14].

Therefore, we used well-described patient groups as well as healthy individuals to compare two standard two-tier test strategies, based on an ELISA (either the C6 ELISA or the SERION ELISA), followed by immunoblot confirmation of equivocal and positive ELISA results. The C6 ELISA measures total immunoglobulin to a recombinant C6 peptide and is currently used in our laboratory. The SERION ELISA measures IgM and IgG to two whole-cell lysates of *B. burgdorferi* sensu lato. It is an improved version compared to the one used by Smismans et al. [8] by the addition of recombinant VlsE for the detection of IgG. The SERION ELISA was selected because it is based on different antigens and it uses VlsE instead of the C6 peptide. VlsE evokes a different antibody response compared to the C6 peptide, since the C6 peptide only becomes available after a conformational change of VlsE when *Borrelia* enters the human body [12, 15]. A third test strategy was also included and consisted of a more unconventional approach based on the combination of both ELISAs as a screening test and immunoblot confirmation of all results, except concordant negative results.

## Materials and methods

### Study population

To qualify for inclusion in this study, all healthy individuals and (hospital) patients had to be ≥18 years old. Healthy individuals, with an increased risk of a tick bite, were recruited in the period between February 2013 and December 2015. Most healthy individuals consisted of personnel of the Diakonessenhuis Hospital or the St. Antonius Hospital, both located in the centre of the Netherlands, close to forested areas. In the same period, Boy Scout patrol leaders, owners of hunting dogs, recreational runners and personnel of the National Institute for Public Health and the Environment (RIVM), all with recreational activities in high-risk areas for tick bites, were asked to participate. Healthy individuals who mentioned antibiotic treatment for Lyme borreliosis in the past in their questionnaire were included in a separate group, referred to as treated healthy individuals. Lyme neuroborreliosis patients enrolled in this study had to fulfil at least two of the following criteria, as proposed by the European Federation of Neurological Societies (EFNS): (i) the presence of neurological symptoms suggestive of Lyme neuroborreliosis without other obvious explanations, (ii) cerebrospinal fluid (CSF) pleocytosis (≥5 leucocytes/μL) and/or (iii) *Borrelia*-specific intrathecal antibody production. A patient was diagnosed with definite Lyme neuroborreliosis when all three criteria were met and with possible Lyme neuroborreliosis when two criteria were fulfilled [16] and they had either been treated for Lyme neuroborreliosis in the past or were recently diagnosed with (active) Lyme neuroborreliosis. For inclusion in the study, intrathecal IgM/IgG was determined by the second-generation IDEIA™ Lyme Neuroborreliosis test (Oxoid, Cambridgeshire, United Kingdom). Patients who had been diagnosed and treated for Lyme neuroborreliosis between February 2004 and September 2012 were enrolled from March 2013 to March 2015; active Lyme neuroborreliosis patients were recruited from December 2010 to December 2015 and were only included if they had not yet started antibiotic treatment for Lyme neuroborreliosis. Active Lyme neuroborreliosis patients who had finished antibiotic treatment could also be included as treated Lyme neuroborreliosis patients when at least one year had passed after their inclusion as an active Lyme neuroborreliosis case.

All healthy individuals and patients were asked to fill in a Lyme-specific questionnaire, which included questions regarding tick bites, presence of erythema migrans (EM), antibiotic treatment for Lyme borreliosis and (self-reported) complaints at the moment of inclusion and during possible earlier episodes of Lyme borreliosis. Information regarding the clinical symptoms, pleocytosis, intrathecal antibody production and the clinical outcome (in case of treated Lyme neuroborreliosis patients) was extracted from the hospital information system. Patients were considered to have a good recovery when, within six months after antibiotic treatment finished, symptoms were absent or had considerably decreased. Patients were considered as treatment failure if severe symptoms continued for >6 months after they had finished antibiotic treatment. Healthy individuals were only recruited when they reported no complaints at the moment of inclusion.

### Serology

Serum samples of all study subjects were tested in two ELISAs and one immunoblot. All tests were performed according to the manufacturer’s instructions using a DS2 automated ELISA instrument (Dynex® DS2, Dynex Technologies, Chantilly, VA, USA) and analysed with the DS-Matrix™ software (Dynex Technologies). An ELISA result was called reactive when the result was equivocal or positive [5]. When both IgG and IgM were determined separately (SERION ELISA and immunoblot), the final result was based on a combination of the results of both immunoglobulins; negative when both IgM and IgG were negative, equivocal when at least one of these was equivocal and positive when at least one of these was positive. When immunoblot confirmation was performed, this result determined the final serology result, independent of an equivocal or positive ELISA result. The C6 ELISA was performed immediately after blood sampling. Immunoblot confirmation was done ≤2 weeks after blood sampling for samples with C6 ELISA index scores between 0.91 to 3.00, according to the protocol of our laboratory. For the purpose of this study, the SERION ELISA was performed on all samples several months to years later. In addition, the samples with C6 ELISA index scores ≥3.00 and any additional reactive SERION ELISA/negative C6 ELISA results were confirmed with immunoblot. Blood samples were stored at 4 °C for two weeks after blood sampling and at −20 °C for longer storage.

#### C6 ELISA

The C6 ELISA (Immunetics, Boston, MA, USA) is based on a synthetic C6 peptide, which is derived from a highly immunogenic part (invariable region 6) of the VlsE lipoprotein [13].

#### SERION ELISA

The SERION ELISA IgM and IgG tests (SERION ELISA classic *Borrelia burgdorferi* IgM and IgG, Institute Virion/Serion GmbH, Würzburg, Germany) are both based on a combination of bacterial lysates of *B. afzelii* P-Ko [17] and *B. garinii* [18]. For IgG detection, the lysates are enriched with recombinant VlsE.

#### Immunoblot

RecomLine IgM and IgG strips (Mikrogen GmbH, Neuried, Germany) containing purified recombinant *B. burgdorferi* sensu lato antigens (OspA, OspC, p100, VlsE, p39, p58 and p18) were used [19]. The results were measured with an automated recomScan system using the recomScan Software (Mikrogen GmbH).

### Test strategies

In this study, three different test strategies were compared. The first two strategies were based on the recommended two-tier test strategy; a screening ELISA followed by immunoblot (IB) confirmation of equivocal or positive ELISA results [1]. The C6 ELISA was used as a screening assay in the first strategy (C6/IB strategy) and the second strategy used the SERION ELISA as a screening test (SE/IB strategy). The third strategy was based on the two ELISAs followed by immunoblot confirmation on all combinations of results, except concordant negative results (SE + C6/IB strategy).

### Statistical analysis

Dichotomous, unrelated samples were analysed using Pearson’s Chi-squared test or Fisher’s exact test. Post-hoc tests consisted of two-group comparisons using Pearson’s Chi-squared test or Fisher’s exact test. The non-parametric Cochran’s Q test for >2 related samples was used for comparison of the ELISA and strategy results and post-hoc tests consisted of the McNemar’s test. For these statistical analyses, equivocal results were combined with positive results. Quantitative data were analysed using the Kruskal–Wallis test for >2 group comparisons. Two-group comparisons and post-hoc tests consisted of the Mann–Whitney test. A *p*-value <0.05 was interpreted as statistically significant, unless Bonferroni correction was applied; Bonferroni correction was applied to all post-hoc tests. Concordance was determined between the C6 ELISA and the SERION ELISA and between the C6/IB strategy and the SE/IB strategy; concordance was calculated as the number of matching positive, equivocal and negative results compared to the overall results within a group. Correlations were calculated using Spearman’s correlation coefficient (r_s_). Logistic regression was applied to calculate the contribution of various variables that could cause discordant test results. The IBM SPSS software package (version 21) was used (Armonk, NY, USA).

## Results

### Study population

#### (Treated) Healthy individuals

A total of 174 healthy individuals were included in this study and the median age at inclusion was 42.3 years (interquartile range (IQR): 28.0–53.4). Twenty-seven of 174 (15.5%) healthy individuals reported antibiotic treatment for Lyme borreliosis in the past (median of 5.0 years ago; IQR 2.0–7.0) and were, therefore, classified as treated healthy individuals; 22/27 (81.5%) reported having had an EM, 4/27 (14.8%) reported a diffuse redness after a tick bite and 1/27 (3.7%) had flu-like symptoms after a tick bite (Table 1). The median age at inclusion of the treated healthy individuals was 53.1 years (IQR: 38.3–57.6). The remaining 147/174 (84.5%) individuals were classified as healthy individuals and had a median age of 40.9 years (IQR: 27.0–51.8) at inclusion and were younger than the other three groups (*p* ≤ 0.001) (Table 1).

**Table 1:** Demographic and clinical characteristics of the four different groups in this study

Variable	Healthy individuals (*n* = 147)	Treated healthy individuals (*n* = 27)	Treated LNB patients^b^ (*n* = 36)	Active LNB patients^b^ (*n* = 27)	*p*-value overall	*p*-value two-group^d^
Sex; no. of males (%)	57 (38.8)	12 (44.4)	19 (52.8)	14 (51.9)	0.339	NA
Age (at inclusion); median, years (IQR)	40.9 (27.0–51.8)	53.1 (38.3–57.6)	59.1 (49.4–66.2)	57.8 (47.8–72.8)	<0.001	≤0.001^e^
Tick bite (%)	86 (58.5)	25 (92.6)	27 (75.0)	9 (56.3)^c^	0.003	≤0.008^f^
EM (%)	4 (2.7)	22 (81.5)	9 (25.0)	4 (25.0)^c^	<0.001	≤0.004^g^
Time of inclusion after AB treatment finished; median, years (IQR)	NA	5.0 (2.0–7.0)^a^	6.1 (3.5–8.4)	NA	NA	0.071
Pleocytosis (before AB treatment started); median (IQR)	NA	NA	52.0 (21.0–113.5)	112.0 (33.0–214.0)	NA	0.050
Definite LNB; *n* (%)	NA	NA	30 (83.3)	22 (81.5)	NA	0.553
Possible LNB based on clinical symptoms and:	NA	NA				
Pleocytosis; *n* (%)			1 (2.8)	4 (14.8)		
Intrathecal antibody production; *n* (%)			5 (13.9)	1 (3.7)		
Recovery status^h^						
Good recovery; *n* (%)			27 (81.8)			
Treatment failure; *n* (%)			6 (18.2)			

LNB: Lyme neuroborreliosis; EM: erythema migrans; IQR: interquartile range; AB: antibiotic; NA: not applicable

^a^One treated healthy individual who did not know when antibiotic treatment took place was excluded

^b^Eight active Lyme neuroborreliosis patients were also included as treated Lyme neuroborreliosis patients

^c^Eleven active Lyme neuroborreliosis patients did not fill out the Lyme-specific questionnaire and were, thus, excluded from the calculations

^d^For all two-group comparisons with a significant difference, the Bonferroni correction was applied (*p*: 0.050/6 = 0.008)

^e^Significant difference in age at inclusion for healthy individuals compared to the remaining three groups

^f^Significant difference in percentage of tick bites for treated healthy individuals compared to healthy individuals and active Lyme neuroborreliosis patients

^g^Significant difference in percentage of erythema migrans for healthy individuals compared to the remaining three groups and for treated healthy individuals compared to treated and active Lyme neuroborreliosis patients

^h^The clinical outcome could not be established for three treated Lyme neuroborreliosis patients

#### Treated Lyme neuroborreliosis patients

Thirty-six treated Lyme neuroborreliosis patients were included and their median age was 59.1 years (IQR: 49.4–66.2) (Table 1). Most treated Lyme neuroborreliosis patients had been diagnosed with radiculopathy (*n* = 24; 66.7%) or cranial nerve paresis (*n* = 17; 47.2%), such as facial nerve paralysis (nerve VII) or abducens nerve palsy (nerve VI). Other diagnoses included meningitis (*n* = 2; 5.6%) and peripheral neuropathy (*n* = 3; 8.3%). Seven patients with radiculopathy also suffered from facial nerve paralysis, one also had meningitis. One patient had been diagnosed with facial nerve paralysis and peripheral neuropathy. Treated Lyme neuroborreliosis patients were included approximately 6.1 years (IQR: 3.5–8.4) after antibiotic treatment finished and treatment consisted of intravenous ceftriaxone for 14 or 30 days; however, one patient switched to doxycycline (for 14 days) after 10 days due to an allergic reaction. As many as 33/36 (91.7%) of the treated Lyme neuroborreliosis patients had a known clinical outcome; 27 (81.8%) of them had a good recovery and six (18.2%) of them were considered a treatment failure (Table 1).

#### Active Lyme neuroborreliosis patients

Twenty-seven active Lyme neuroborreliosis patients were included and their median age was 57.8 years (IQR: 47.8–72.8) (Table 1). Most active Lyme neuroborreliosis patients were diagnosed with radiculopathy (*n* = 8; 29.6%) or cranial nerve paresis (*n* = 7; 25.9%), such as facial nerve paralysis or abducens nerve palsy. Other diagnoses included meningitis (*n* = 5; 18.5%) or peripheral neuropathy (*n* = 2; 7.4%). Four active Lyme neuroborreliosis patients with radiculopathy also suffered from facial nerve paralysis, one patient had facial nerve paralysis and meningitis, and one patient had radiculopathy as well as peripheral neuropathy. Eight of 27 (29.6%) active Lyme neuroborreliosis patients were, at a later moment, also included as a treated Lyme neuroborreliosis patient and were, thus, included in both groups. The median time between the two blood sampling moments (and, thus, the time between their inclusion as an active and a treated case) was 2.1 years (IQR: 1.4–2.5). Hence, in total, 63 blood samples originating from 55 Lyme neuroborreliosis patients were investigated in this study.

### ELISA results

**Fig. 1:**
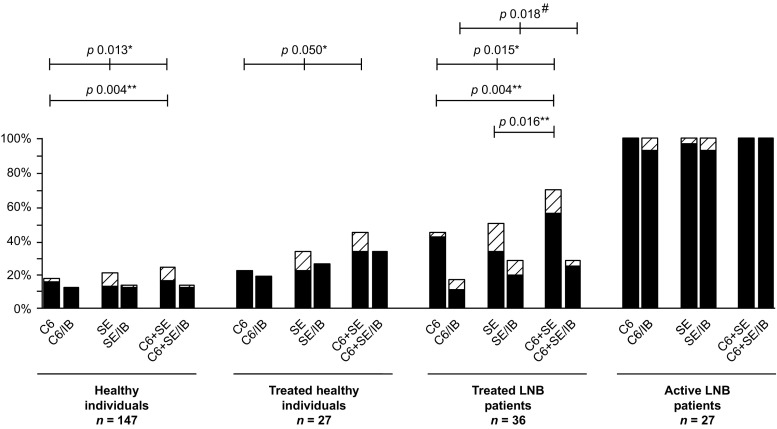
Overall view of the proportions of reactive (equivocal and positive) enzyme-linked immunosorbent assay (ELISA) and test strategy results. LNB: Lyme neuroborreliosis. The *black bars* represent positive results and the *shaded bars* represent equivocal results. C6: C6 ELISA; C6/IB: C6 ELISA plus immunoblot strategy; SE SERION ELISA; SE/IB: SERION ELISA plus immunoblot strategy; SE + C6: combination of SERION ELISA and C6 ELISA; SE + C6/IB: SERION ELISA, C6 ELISA plus immunoblot strategy. The C6 + SE bars represent the percentage of reactive results for at least one of the tests (i.e. C6 ELISA, SERION IgM ELISA or SERION IgG ELISA). Only statistical significant differences are displayed. */#: Significant difference between the ELISAs (*) and the test strategies (#) in the overall dataset; **: Significant difference between the two-group comparisons of the ELISAs. For all two-group comparisons with a significant difference, the Bonferroni correction was applied (*p*: 0.050/3 = 0.017)

**Fig. 2:**
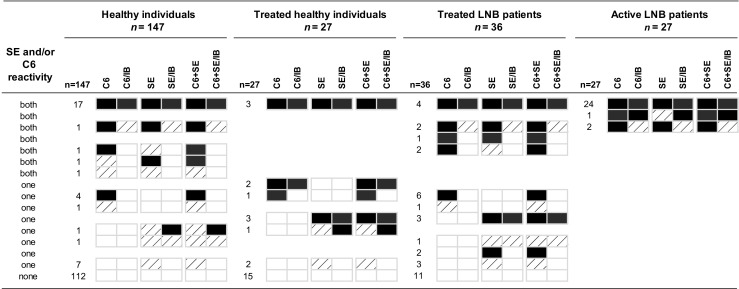
Detailed view of the different ELISA and test strategy results. LNB: Lyme neuroborreliosis. The *black boxes* represent positive results, the *shaded boxes* represent equivocal results and the *white boxes* represent negative results. C6: C6 ELISA; C6/IB: C6 ELISA plus immunoblot strategy; SE: SERION ELISA; SE/IB: SERION ELISA plus immunoblot strategy; SE + C6: combination of SERION ELISA and C6 ELISA; SE + C6/IB: SERION ELISA, C6 ELISA plus immunoblot strategy

All 27 (100%) active Lyme neuroborreliosis patients had reactive results for both ELISAs, but the ELISA results of the other three groups showed more diversity (Fig. 1). In these three groups, the SERION ELISA resulted in more reactive cases compared to the C6 ELISA (24 vs. 15), but the difference within each group was not statistically significant (Fig. 2). The percentage of reactive ELISA results, when both ELISAs were combined, was 23.8% (35/147) for healthy individuals, 44.4% (12/27) for treated healthy individuals and 69.4% (25/36) for treated Lyme neuroborreliosis patients (Fig. 2). For both healthy individuals and treated Lyme neuroborreliosis patients, this percentage was significantly higher than the percentage of reactive C6 ELISA results alone (26/147 (17.7%) and 16/36 (44.5%), respectively) (*p* ≤ 0.004 for both) (Figs. 1 and 2). For treated Lyme neuroborreliosis patients, the percentage of reactive results of the C6 ELISA and the SERION ELISA combined (25/36; 69.4%) was also significantly higher than the percentage of reactive results of the SERION ELISA alone (18/36; 50.0%) (*p* = 0.016) (Figs. 1 and 2).

### Concordance of ELISA results

**Fig. 3:**
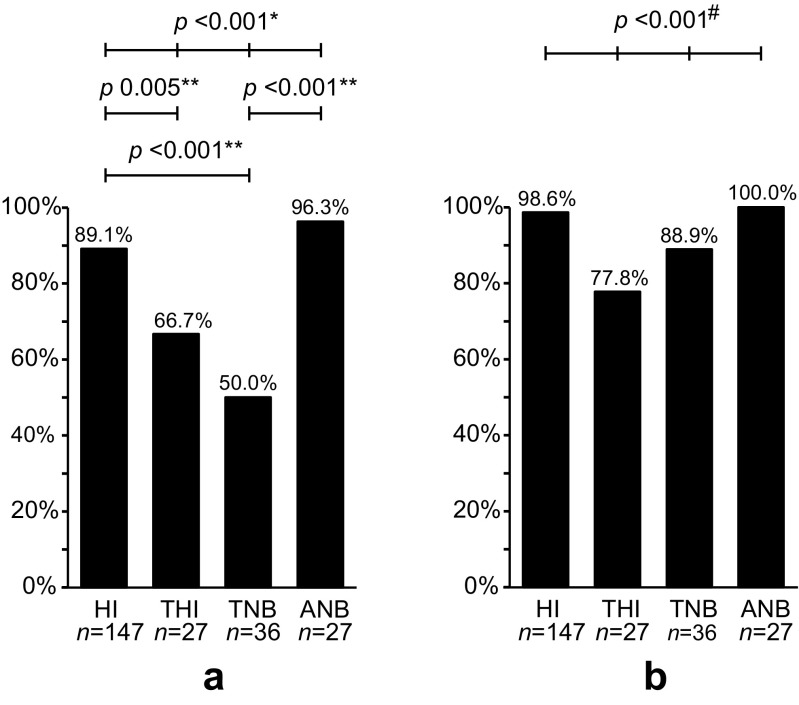
The concordance between the results of the C6 ELISA and the results of the SERION ELISA (**a**) and the concordance between the results of the C6 ELISA plus immunoblot strategy and the SERION ELISA plus immunoblot strategy (**b**). HI: healthy individuals; THI: treated healthy individuals; TNB: treated Lyme neuroborreliosis patients; ANB: active Lyme neuroborreliosis patients; n: number of study subjects. Only statistical significant differences are displayed. For all two-group comparisons with a significant difference, the Bonferroni correction was applied (*p*: 0.050/6 = 0.008). */#: Significant difference between the ELISAs (*) and the test strategies (#) in the overall dataset; **: Significant difference between the two-group comparisons of the ELISAs

The concordance between both ELISAs was lower for treated healthy individuals (18/27; 66.7%) and treated Lyme neuroborreliosis patients (18/36; 50.0%) than for healthy individuals (131/147; 89.1%) and active Lyme neuroborreliosis patients (26/27; 96.3%) (*p* ≤ 0.005) (Fig. 3a). The majority of the concordant ELISA results were based on negative ELISA results, except for active Lyme neuroborreliosis patients, for whom only reactive ELISA results were found. In contrast, 112/131 (85.5%) ELISA results among healthy individuals were concordant negative (Fig. 2), of whom only three (2.3%) reported a previous (untreated) EM (data not shown). Although it was unclear whether healthy individuals had been infected with *Borrelia* in the past, the percentage of concordant negative results among healthy individuals did not differ from the percentage of concordant negative results among treated healthy individuals or treated Lyme neuroborreliosis patients (15/18 (83.3%) and 11/18 (61.1%), respectively) (Fig. 2).

### Immunoblot results

In total, 99 immunoblots had to be performed based on 44 (44.4%) discordant and 55 (55.6%) concordant ELISA results (Fig. 2). All 27 (100%) active Lyme neuroborreliosis patients were confirmed by immunoblot analysis. A lower percentage of confirmed cases was found among the other three groups. Of the 35 healthy individuals that had a reaction with one or both ELISAs, 20 (57.1%) were confirmed by immunoblot; among treated healthy individuals, this was 75.0% (9/12) and among treated Lyme neuroborreliosis patients, it was 40.0% (10/25) (Fig. 2).

With regard to the 55 concordant reactive ELISA results, 53 (96.4%) were confirmed by immunoblot. All 53 had a positive result for the C6 ELISA and the SERION IgG ELISA, of which 31 (58.5%) also had an equivocal or positive result for the SERION IgM ELISA. Immunoblot confirmation was based on an IgG response against at least two antigens; IgG against VlsE was found in all 53 cases, followed by IgG against p41 flagellin (39/53; 73.6%) and subsequently by IgG against p100, p18, p58, p39 and OspC (range 23 to 9; 43.3% to 17.0%) (data not shown). Only 16/53 (30.2%) cases were also confirmed based on the presence of IgM, of which most cases were found among active Lyme neuroborreliosis patients (13/16; 81.3%). For all 16 IgM confirmed cases, antibodies were found against OspC, followed by IgM against p41 flagellin (11/16; 68.8%) (data not shown). The remaining 2/55 (3.6%) concordant reactive ELISA results were not confirmed. One case was a healthy individual with an equivocal result for both the C6 ELISA and the SERION IgM ELISA, and the other case was a treated Lyme neuroborreliosis patient with a positive result for both the C6 ELISA and the SERION IgM ELISA (Fig. 2).

Analysis of the 44 discordant ELISA results showed that most cases were SERION ELISA reactive/C6 ELISA negative (*n* = 24; 54.5%) (Fig. 2). Those cases were based on reactivity for the SERION IgM ELISA alone (*n* = 16/24; 66.6%), for the SERION IgG ELISA alone (3/16; 18.8%) or for both the SERION IgM and the SERION IgG ELISAs (5/16; 31.3%). In total, 13/44 (29.5%) discordant ELISA results were confirmed, of which only two were based on C6 ELISA reactive/SERION ELISA negative results. Interestingly, none of the 15 C6 ELISA reactive/SERION ELISA negative results showed a VlsE band. Immunoblot confirmation of the two cases was based on the presence of IgM against OspC (*n* = 2) and p41 flagellin (*n* = 1) (data not shown). In total, 10/24 (41.7%) SERION ELISA reactive/C6 ELISA negative results were confirmed. Five (31.3%) out of the 16 solitary IgM reactive cases were confirmed based on IgM against OspC (*n* = 5) and p41 flagellin (*n* = 1); one also had IgG against OspC and p41 flagellin. One (33.3%) out of the three solitary IgG cases was confirmed based on IgG against p41 flagellin and p18, and 4/5 (80.0%) IgM and IgG reactive cases were confirmed. Two cases had IgM against OspC and p41 flagellin, of whom the second case also had IgG against VlsE and p18, the third case had IgG against OspC and p41 flagellin, and the fourth case had IgG against VlsE and p39 (data not shown). The remaining confirmed case was an active neuroborreliosis patient who had reactive—but discordant—ELISA results. Immunoblot analysis showed IgG against VlsE, p41 flagellin and p39 (data not shown).

### Concordance of test strategy results

For all groups, the concordance between the C6/IB strategy and the SE/IB strategy was higher than the concordance found between both ELISAs. Only for treated healthy individuals was the concordance still significantly lower than for healthy individuals (21/27 (77.8%) and 145/147 (98.6%), respectively) (*p* < 0.001) (Fig. 3b). Interestingly, no correlation was found between the C6 ELISA reactive/SERION ELISA negative results and the immunoblot results (r_s_ = 0.154; *p* = 0.584), but a correlation was found between the SERION ELISA reactive/C6 ELISA negative results and the immunoblot results (r_s_ = 0.555; *p* = 0.005). Thus, reactive SERION ELISA results were more often confirmed by immunoblot analysis than the reactive C6 ELISA results.

### Possible explanations for the discordant ELISA and test strategy results

Discordant ELISA results are caused by variability in the amount and type of antibodies, which, in turn, may be influenced by antibiotic treatment and/or the natural course of clearance of infection. The natural course of clearance of infection may be influenced by the age, sex or recovery of the patient, whether or not a tick bite or EM were observed, the time between end of antibiotic treatment and blood sampling and duration of symptoms before the start of antibiotic therapy. We determined the possible contribution of these factors to the discordance of test results. Analysis of all study participants showed that only antibiotic treatment was strongly associated with discordant ELISA and discordant test strategy results [odds ratio (OR): 10.52; *[* < 0.001 and OR: 9.98; *p* = 0.014, respectively] (Table 2). Among healthy individuals and Lyme neuroborreliosis patients who were both treated, the time between the end of antibiotic treatment and blood sampling did not contribute to an increased discordance (data not shown). However, solely among treated Lyme neuroborreliosis patients, the C6 ELISA index scores did decrease with increasing time between the end of antibiotic treatment and blood sampling (r_s_ = −0.408; *p* = 0.013). For the SERION IgM and SERION IgG ELISAs, and among treated healthy individuals, we did not find such a correlation (data not shown). When both treated groups were analysed separately, older age was associated with a slight increase in the percentage of discordant ELISA results among treated Lyme neuroborreliosis patients (OR: 1.10; *p* = 0.015). In contrast, older age was associated with a lower percentage of discordant test strategy results among treated Lyme individuals, although only slightly (OR: 0.82; *p* = 0.033). There was no association between the recovery status or the duration of symptoms and the percentage of discordant ELISA or test strategy results among treated Lyme neuroborreliosis patients (data not shown).

**Table 2:** Logistic regression model assessing risk factors for discordant test results among study participants

Test	Parameter	B	SE	Wald	df	*p*-Value	Odds ratio	95% CI for OR	
								Lower	Upper
ELISAs	Sex (male)	−0.15	0.37	0.17	1	0.684	0.86	0.42	1.78
	Tick bite (yes)	−0.49	0.42	1.38	1	0.240	0.61	0.27	1.39
	EM (yes)	−0.65	0.50	1.70	1	0.193	0.52	0.19	1.39
	Antibiotic treatment (yes)	2.35	0.49	23.49	1	<0.001^a^	10.52	4.06	27.26
	Age	0.01	0.01	0.21	1	0.647	1.01	0.98	1.03
Test strategies	Sex (male)	−0.47	0.64	0.54	1	0.462	0.62	0.18	2.20
	Tick bite (yes)	−0.83	0.74	1.26	1	0.262	0.44	0.10	1.86
	EM (yes)	0.78	0.72	1.15	1	0.283	2.17	0.53	8.97
	Antibiotic treatment (yes)	2.30	0.93	6.10	1	0.014^a^	9.98	1.61	61.92
	Age	−0.02	0.02	0.94	1	0.333	0.98	0.93	1.02

The logistic regression model gives the probability that a discordant test result will be found (*n* = 226; 11 active Lyme neuroborreliosis patients did not fill out the Lyme-specific questionnaire and were, thus, excluded from the calculations). B: coefficient for the constant; SE: standard error; df: degrees of freedom for the Wald Chi-square test; CI: confidence interval; OR: odds ratio

^a^Statistically significant

## Discussion

In this study, we compared two standard two-tier test strategies by using two screening ELISAs and a conformational immunoblot on various well-defined Dutch Lyme patients and healthy individuals. High concordances between the results of the test strategies were found for healthy individuals and active Lyme neuroborreliosis patients groups (range 98.6–100%); however, low concordances were observed for Lyme neuroborreliosis patients and healthy individuals who had been treated for Lyme borreliosis in the past (range 77.8–88.9%). Discordant test results represent variability in the amount and type of antibodies, which, in turn, may be influenced by antibiotic treatment and/or the natural course of clearance of infection. Of the investigated factors affecting the natural clearance of the infection, only age contributed to discordant ELISA or test strategy results within both treated groups, but only to a minimal extent. Older age was associated with an increase of discordant ELISA results among treated Lyme neuroborreliosis patients and with a decrease of discordant test strategy results among treated healthy individuals. The significantly higher discordance between the ELISA results in the two treated groups compared to the almost concordant ELISA results in active Lyme neuroborreliosis patients (96.3%) and healthy individuals (89.1%) was associated to antibiotic treatment against Lyme borreliosis. This suggests that antibiotic treatment influences the pace at which the detected serum antibodies wane.

Furthermore, this study showed that the SERION ELISA had more positive results than the C6 ELISA and also led to a higher percentage of immunoblot-confirmed results. This finding implies that serum antibodies against the C6 peptide wane faster than other *Borrelia*-specific serum antibodies. In this study, we found a significant correlation among treated Lyme neuroborreliosis patients between the decrease in C6 ELISA index scores and a longer time between the end of antibiotic treatment and blood sampling. Although the number of cases in our study is small, other studies also showed a decline of C6 antibodies and a >4-fold decrease in C6 ELISA results within one year after antibiotic treatment [20, 21]. In 9/13 (69.2%) immunoblot-confirmed sera, for which discordant ELISA results were found, the presence of IgM against OspC, and in some cases p41 flagellin, was found. In 10/13 cases (76.9%), confirmation was based on a reactive SERION ELISA result only. Since both OspC and p41 flagellin are part of the whole-cell lysate used in the SERION ELISA, this might explain the higher positive rate for this ELISA. Aguero-Rosenfeld et al. [22] also reported the persistence of IgM against OspC and p41 flagellin in more than half of the patients in their study during a follow-up at one year. The persistence of the immune response against OspC and p41 flagellin up to 20 years after successful treatment has also been described by Kalish et al. [4]. To emphasise, the presence of *Borrelia*-specific serum antibodies is no proof of an active Lyme borreliosis infection or proof of a false-positive reaction of the ELISA used, but could well be explained by a previous, cleared Lyme borreliosis infection, with or without antibiotic treatment.

Surprisingly, all 15 reactive C6 ELISA results with negative SERION ELISA results failed to show a VlsE band in the immunoblot analysis. A C6 ELISA positivity could be expected, since 10/15 (66.7%) cases were treated for Lyme borreliosis in the past, but the absence of a VlsE band for all cases was unexpected. It could be explained by a reduced sensitivity of the immunoblot [23] or by a difference in the accessibility of the C6 peptide on the immunoblot. The C6 peptide is not exposed on the surface of VlsE and becomes available only when VlsE is processed after a *Borrelia* infection has occurred and it is, therefore, suggested that C6 elicits a different antibody response compared to VlsE, which is supported by this study [12, 15].

Although this study underlines the importance of the two-tier test strategy, the analysis of the third strategy, SE + C6/IB, was also of interest, as this strategy resulted in the highest number of sera for which *Borrelia*-specific serum antibodies were detected. A disadvantage was the high number of immunoblot tests that were needed to get the final results. The strategy could be simplified by not confirming concordant positive ELISA results with immunoblot. This simplified SE + C6/IB would substantially decrease the number of immunoblots needed from 99 to 45 (−54.5%), which is also less compared to the C6/IB strategy (45 vs. 75 (−40.0%)) or the SE/IB strategy alone (45 vs. 84 (−46.4%)). The final results would almost be similar, as only one treated neuroborreliosis patient would be recorded differently (both ELISAs positive/immunoblot negative). Since the immunoblot is more labour-intensive, expensive and the interpretation more subjective compared to an ELISA, this simplified SE + C6/IB strategy is interesting, as other studies also underline the potential of a two-ELISA strategy for the diagnosis of Lyme borreliosis [24, 25].

The selection criteria used in our study ruled out the inclusion of other than Lyme neuroborreliosis patients. Since Lyme neuroborreliosis is the most common clinical presentation of disseminated Lyme borreliosis in the Netherlands and the criteria for Lyme neuroborreliosis, as proposed by the EFNS [16], are clear and easy to apply, this ensured the inclusion of true Lyme neuroborreliosis cases. A potential bias could be the lower age of the healthy individuals compared to the other three groups. Furthermore, eight patients were included twice in this study, both as an active Lyme neuroborreliosis patient and later as a treated Lyme neuroborreliosis patient. Nevertheless, we argue that the influence of these potential biases are limited, since they most likely do not affect the performance of the ELISAs and the immunoblot, or, if so, the possible effect should be equal for all tests concerned. Finally, the number of study participants in our study is limited and future studies including more patients and healthy controls, as well as different Lyme manifestations, are needed to strengthen our findings. Future studies could also investigate the effect of the host immune response and the infecting *Borrelia* species on test results.

To conclude, this study showed a lower test agreement in healthy individuals and Lyme neuroborreliosis patients who were both treated for Lyme borreliosis in the past. We showed that antibiotic treatment influences serological test results and that the effect differs for the different assays. Although the number of cases is limited and we did not take into account the possible influence of the different clinical manifestations, we do believe that antibiotics contribute to the variation in the kinetics of the antibodies directed against the different *Borrelia* antigens used in the tests. Although only two commercial ELISAs and one commercial immunoblot were tested, our data suggest that this holds true for other assays based on different antigens and/or different *Borrelia* strains. This study emphasises that care should be taken when Lyme serology is considered and symptoms are non-specific. It supports the general opinion that serological testing for *Borrelia*-specific serum antibodies should only be performed in case of a high a priori chance of Lyme borreliosis. To our knowledge, this is the first study that gives an in-depth insight into the diagnostic challenges which arise when individuals have been treated for Lyme borreliosis in the past.
